# The Moderating Role of Perceived Social Support on the Relationship between Physical Functional Impairment and Depressive Symptoms among Chinese Nursing Home Elderly in Hong Kong

**DOI:** 10.1100/tsw.2011.93

**Published:** 2011-05-05

**Authors:** Sylvia Y. C. L. Kwok, Dannii Y. L. Yeung, Annie Chung

**Affiliations:** ^1^ Department of Applied Social Studies, City University of Hong Kong, Hong Kong; ^2^ Social Service Department, Yuen Yuen Institute, Hong Kong

**Keywords:** perceived institutional peer support, physical functional impairment, perceived family support, elderly depressive symptoms, nursing home elderly

## Abstract

With reference to the stress-buffering model, this study aimed to examine the moderating role of perceived social support (including institutional peer support and family support) on the relationship between physical functional impairment, as a source of stress, and depressive symptoms among Chinese nursing home elderly in Hong Kong. The study used a cross-sectional survey method and convenience sampling. The subjects were recruited from two private nursing homes. A total of 187 elderly (54 males and 133 females) participated in the survey. Interviews were conducted by experienced research assistants. The Geriatric Depression Scale was used to assess depressive symptoms of each participant. Pearson correlational analyses showed that females reported more depressive symptoms than their male counterparts, and a positive relationship was found between education level and depressive symptoms. Perceived institutional peer support was negatively correlated, while physical functional impairment was positively correlated with depressive symptoms. However, there was no significant correlation between perceived family support and depressive symptoms. Hierarchical regression analyses revealed that physical functional impairment and perceived institutional peer support were significant predictors of elderly depressive symptoms, while perceived family support was not a significant predictor, after statistically controlling for the influence of gender and education level. Perceived institutional peer support, but not perceived family support, was found to moderate the negative impact of physical functional impairment on elderly depressive symptoms. The theoretical and practical implications of this study were then discussed.

### INTRODUCTION

The elderly population in Hong Kong is increasing rapidly, with 12.4% of the population currently over the age of 65 and it is projected to increase to 27% by the year 2033[1]. The proportion of older people living in residential care homes increased from 5.5% in 1996 to 10% in 2006[1]. Nursing homes provide residential care, meals, personal care, regular basic medical and nursing care, and social support for the elderly who suffer from poor health and physical or mental disabilities, with deficiency in daily activities, but who are mentally suitable for communal living and are assessed to be in the severe impairment level[2].

Depression is a serious health risk that is prevalent in a nursing home setting. In a study of 245 elderly Chinese private nursing home residents in Hong Kong, significant depressive symptoms were detected in 29% of the subjects[3]. Similarly, about 25% of nursing home residents suffered from mild to severe depression in the U.S.[4]. These statistics show the high prevalence of depression among the institutionalized elderly, inferring a pressing concern to study the correlates of depression among nursing home residents. Previous studies revealed that deterioration of health, lack of social resources, and loss of established social relationships contribute to the high incidence of depression among nursing home residents[5,6]. Thus, the present study aims to use a stress-buffering model to examine the relationship among physical functional impairment, as a source of stress, perceived social support (including institutional peer support and family support), and depressive symptoms among Chinese elderly in privately owned nursing homes in Hong Kong.

#### Stress-Buffering Model

According to the stress-buffering model, stress interacts with social support such that the effects of stress are significantly lessened under conditions of strong social support[7]. Social support is defined as resources provided by others as coping assistance in times of need[8] and is found to be a moderator to buffer against the negative impact of stress. Most of the elderly living in nursing homes have certain levels of functional impairment, and they mainly get support from their family members and peers in the institution. Hence, with reference to the model, it is helpful to examine the moderating roles of perceived family support and institutional peer support in the relationship between physical functional impairment, as a source of stress, and elderly depressive symptoms.

#### Physical Functional Impairment and Elderly Depressive Symptoms

Functional loss has been conceptualized as a chronic stressor that affects an individual's ability to interact with the environment and consequently increases the risk of depression. Studies showed a significantly positive relationship between physical functional impairment and emotional distress[9,10]. Functional disability restricts an individuals' mobility, which in turn impedes their engagement in social relationships and fulfillment of social roles. This weakening of social bonding may increase the risk of depression among older people. Hence, how physical functioning relates to depressive symptoms among Chinese elderly in nursing homes remains to be explored.

#### Family Support and Elderly Depressive Symptoms

Previous research found that family support negatively predicted depression[11,12]. For example, in a cross-sectional study of Chinese elderly, those who received more support from their family members (not living with elders) tended to report less symptoms of depression than did a random community sample of elderly living with their family members[13]. As there may be a difference in the relationship between perceived family support and depressive symptoms for those elderly living and not living with their family members, how perceived family support relates to depressive symptoms among the nursing home residents is worth examining.

### Institutional Peer Support and Elderly Depressive Symptoms

Regarding institutional peer support, studies showed that perceived peer support within the institution and satisfaction with the peer support were negatively correlated with geriatric depression[14]. Studies also found a negative correlation between depression and size of social network, as well as satisfaction with peer support[10,15]. Given that the importance of institutional peer support to the elderly in nursing homes may be different from that of peer support to the community elderly, it is important to study the relationship between perceived institutional peer support and depressive symptoms of institutionalized elders.

### Moderating Role of Social Support on Elderly Depressive Symptoms

Studies on the moderating role of social support were equivocal. One study stated that social support moderated the effect of stressful life events on the psychological well-being of Chinese older adults in Hong Kong[15]. Perceived social support has been found to buffer the negative impact of individual-level stressors on psychological distress and depression among childless elderly in Canada and the U.S.[16]. Specifically, few studies have been conducted on the moderating roles of family support and institutional peer support in depression. One study found that only perceived support from peers, but not from family members, was significantly associated with the psychological well-being of the nursing home patients[14]. However, as Chinese elderly place heavy emphasis on family care and interpret it as a sign of filial piety from the children, they may perceive support from peers as less important[17]. Hence, it would be interesting to study whether perceived institutional peer support and/or family support buffer the relationship between physical functional impairment and depression among nursing home elderly

### Gender and Education as Covariates of Elderly Depressive Symptoms

Gender differences are a common concern in depression research. In a local study, Chow and colleagues[3] found a prevalence rate of depression of 25.3 and 31.2% for elderly males and females residing in nursing homes, respectively. However, a higher proportion of women (female: 33% vs. male: 18.6%) were diagnosed with depression in a Western study of depression among the very old[18]. The effect of gender on depression varies across life spans. Specifically, studies have found that the gender difference emerged during adolescence, stabilized throughout adulthood, and then declined during old age[19]. Previous studies also reported that individuals with better education had significantly better mental health, compared with those with a lower education level[20]. The elderly with higher educational experience may have more recreational activities and hobbies, which might reduce the probability of depression. Previous studies showed that gender and education level of the elderly were significant predictors of depressive symptoms; hence, they were included as covariates in the regression analyses.

### Limitations in the Existing Literature

Existing studies have several gaps. First, they mainly focus on the elderly living in the community. Studies on the depressive symptoms of Chinese elderly living in nursing homes, with particular reference to Chinese culture, are limited. Second, previous research lacks a theoretical framework to explain the relationship among stress, social support, and elderly depressive symptoms. Third, the moderating mechanism of perceived institutional peer support and family support that links physical functional impairment and elderly depressive symptoms in nursing homes has seldom been explored. Fourth, most previous studies were conducted in a Western context. A survey of the PsycINFO database in January 2011 showed that there were 7,226 articles on "elderly" and "depression". However, only 181 articles were related to Chinese studies. As Chinese people constitute roughly one-fifth of the world's population, further research should be conducted on this subject matter[21].

### Research Questions and Hypotheses

Against the above background, several research questions were addressed in the current study. (1) What is the relationship among gender, education, and elderly depressive symptoms? From the results of previous research[3,20], it was hypothesized that female elders would have a higher level of depressive symptoms than male elders (Hypothesis 1a), and the elderly with higher education would a lower level of depressive symptoms (Hypothesis 1b). (2) What is the relationship among physical functional impairment, perceived family support, perceived institutional peer support, and elderly depressive symptoms? Some of the previous findings on depression of community elderly were different from those of the nursing home sample. For example, one study did not find a significant correlation between functional disability and geriatric depression[22]; another study did not find a significant correlation between satisfaction with family support and elderly depression in a community sample[23]. However, with reference to the particular situations of those elders living in nursing homes and based on previous studies[9,10,11,12,14], it was hypothesized that physical functional impairment would have a positive relationship with elderly depressive symptoms (Hypothesis 2a), perceived family support would have a negative relationship with elderly depressive symptoms (Hypothesis 2b), and perceived institutional peer support would have a negative relationship with elderly depressive symptoms (Hypothesis 2c). (3) Would perceived institutional peer support and family support moderate the relationship between physical functional impairment and elderly depressive symptoms? Based on the stress-buffering model and previous research[15,16], it was hypothesized that perceived institutional peer support and/or family support would moderate the relationship between physical functional impairment and elderly depressive symptoms.

## METHODS

### Participants and Procedure

The current study was conducted by convenience sampling. Two privately owned nursing homes in Hong Kong joined the study. According to the admission criteria of elderly to the nursing homes in Hong Kong, all the residents must have been 65 years of age or above at the time of admission. Among the 203 nursing home residents who were interviewed, 16 participants gave incomplete responses; thus, they were excluded from further analysis. The final number of participants in this study was 187 (54 males and 133 females). About one-fifth (23%) of them were aged between 65 and 75 years, 40.1% were aged between 76 and 85 years, and the remaining were over 85 years old. About half of them (45.5%) were illiterate and 46.5% had primary education.

The managerial staffs of the nursing homes were first informed about the objectives of this study and the procedures for conducting the interviews. The elderly who scored 18 points or above on the Mini-Mental State Examination (MMSE) were invited to participate in the survey because of their ability to understand the content of the questionnaire. The invited elderly consented to participate in the study, even without any monetary reward. Research assistants who were adequately trained and experienced in conducting face-to-face interviews with older people conducted the interviews. Each interview lasted for about 25 min.

### Measures

- *Depressive symptoms* — The Geriatric Depression Scale[24] consists of 15 items and has been widely adopted in previous studies[25,26]. The current study adopted the Chinese Geriatric Depression Scale (CGDS) to measure the depressive symptoms of nursing home residents[27]. Participants were asked whether they had experienced depressive symptoms in the past 2 weeks. They rated these items on a dichotomous scale (0 = no, 1 = yes) to indicate the presence or absence of each depressive symptom. A sum score was computed, ranging from 0 to 15 symptoms. Higher scores represent more depressive symptoms. The internal consistency of this scale was satisfactory, with a Cronbach's alpha of 0.68.
- *Physical functional impairment* — Functional abilities were measured by Katz's Basic Activities of Daily Living Scale[28]. It consists of six items to assess one's ability to handle daily activities; namely, bathing, dressing, toileting, transferring, continence, and feeding. Participants rated these items on a 3-point scale to indicate their degree of performing the activity, with higher scores representing poorer physical functioning (1 = perform independently, 2 = need some assistance, 3 = have complete dependence). The internal consistency of this scale was high, with a Cronbach's alpha of 0.84. A higher total score indicates poorer physical ability of the respondent.
- *Perceived institutional peer support* — Four items were selected and modified from Lubben's Social Network Scale[29] to reveal the level of perceived peer support in the nursing home. Among the sample items were "How many residents in your nursing home in whom you directly confide your personal matters and feelings (1 = none to 4 = 3 persons or more)?" and "When you have to make an important decision, do you have someone in the nursing home whom you can talk to about it (1 = never to 4 = always)?" The Cronbach's alpha was 0.84. A higher score indicates greater perceived institutional peer support.
- *Perceived family support* — Six items were selected and modified from Lubben's Social Network Scale[29] to assess the level of perceived support from family members. Among the sample items were "How many relatives do you see or hear from at least once a month (1 = none to 4 = 3 persons or more)?" and "When you have an important decision to make, do you have someone in your family whom you can talk to about it (1 = none to 4 = always)?" A higher score indicates greater perceived family support. The Cronbach's alpha of this measure was 0.81.
- *Demographic variables* — The sex and education level (1 = illiterate, 2 = primary education, 3 = secondary education or above) of the participants were incorporated as covariates in the regression analyses of the present study.

## RESULTS

### Depressive Symptoms of the Sample

Results showed that the level of depressive symptoms was fairly low in the current sample of nursing home residents ($M$ = 5.48; $SD$ = 2.10). About 17.6% had a CGDS score of 8 or above, the cut-off point for the presence of a manifest level of depression. T-test analyses showed a significant difference in perceived institutional peer support between the depressed and nondepressed samples ($t(1,185) = 9.15$, $p < 0.001$); however, no significant differences in physical impairment and perceived family support were found.

### Correlation between Major and Demographic Variables

Pearson correlation analyses were performed. Female participants reported more depressive symptoms than their male counterparts ( $r$= -0.18, $p < 0.05$). A positive relationship existed between education level and depressive symptoms ($r$ = 0.25, $p$ < 0.001), implying that elders with a higher education level had more depressive symptoms than those who were illiterate or less educated. Sex and education level were therefore statistically controlled for in the following regression analyses as covariates. However, perceived family support did not correlate with depressive symptoms, physical functional impairment, and perceived institutional peer support (see Table 1).

**TABLE 1**
**Descriptive and Correlation Analyses of Major Variables**

| | | Mean (SD)/Percent | 1 | 2 | 3 | 4 | 5 | 6 |
|---|---|---|---|---|---|---|---|---|
| 1. | Sex[1] | 71.1% | — | | | | | |
| 2. | Education[2] | 1.63 (0.63) | -0.17* | — | | | | |
| 3. | Perceived family support | 16.75 (2.31) | -0.05 | -0.00 | — | | | |
| 4. | Perceived institutional peer support | 17.39 (3.04) | 0.15* | -0.15* | -0.12 | — | | |
| 5. | Physical functional impairment | 13.18 (3.04) | -0.07 | 0.24** | 0.08 | -0.10 | — | |
| 6. | Elderly depression | 5.48 (2.10) | -0.18* | 0.25*** | 0.08 | -0.66*** | 0.18* | — |

*Note*: Sex[1]: 1 = female, 2 = male; Education[2]: 1 = illiterate, 2 = primary education, 3 = secondary education or above. *$p$ < 0.05; **$p$ < 0.01; ***$p$ < 0.001.

Significant associations emerged among perceived institutional peer support, physical functional impairment, and depressive symptoms. Perceived institutional peer support was negatively correlated with depressive symptoms ($r$ = -0.66, $p$ < 0.001), whereas physical functional impairment was positively correlated with depressive symptoms ($r$ = 0.18, $p$ < 0.05). Participants with less perceived institutional peer support and poorer physical abilities tended to have more depressive symptoms (see Table 1).

### Moderating Effect of Perceived Institutional Peer Support and Family Support

Hierarchical multiple regression analyses were performed to determine the moderating effect of perceived institutional peer support and family support. Demographic variables (including sex and education level), then physical functional impairment, perceived institutional peer support and family support, and finally the interaction terms between physical functional impairment and perceived institutional peer support, and between physical functional impairment and perceived family support, were entered into hierarchical blocks. Results showed that physical functional impairment × perceived institutional peer support interaction was significant ($B$ = -0.16, $p$ < 0.01), whereas physical functional impairment × perceived family support interaction was not significant. Perceived institutional peer support was found to buffer the negative impact of physical functional impairment on elderly depressive symptoms. In plotting the graph, perceived institutional peer support was split into two groups according to the median, with a score of 17 or below categorized as low support group and a score higher than 17 as high support group (see Fig. 1). Findings also revealed that gender ($B$ = -0.14, $p$ < 0.05), education level ($B$ = 0.23, $p$ < 0.01), physical functional impairment ($B$ = 0.11, $p$ < 0.05), and perceived institutional peer support ($B$ = -0.62, $p$ < 0.001) were significant predictors of elderly depressive symptoms, whereas perceived family support was not a significant predictor (see Table 2). Since prior studies had reported gender differences in depressive symptoms, additional regression analyses were performed to examine the interaction effect of gender with physical impairment, perceived family support, and institutional peer support on depressive symptoms. Results showed that all interactions were not significant, suggesting that the effect of physical impairment and perceived social support on depressive symptoms did not vary between males and females.

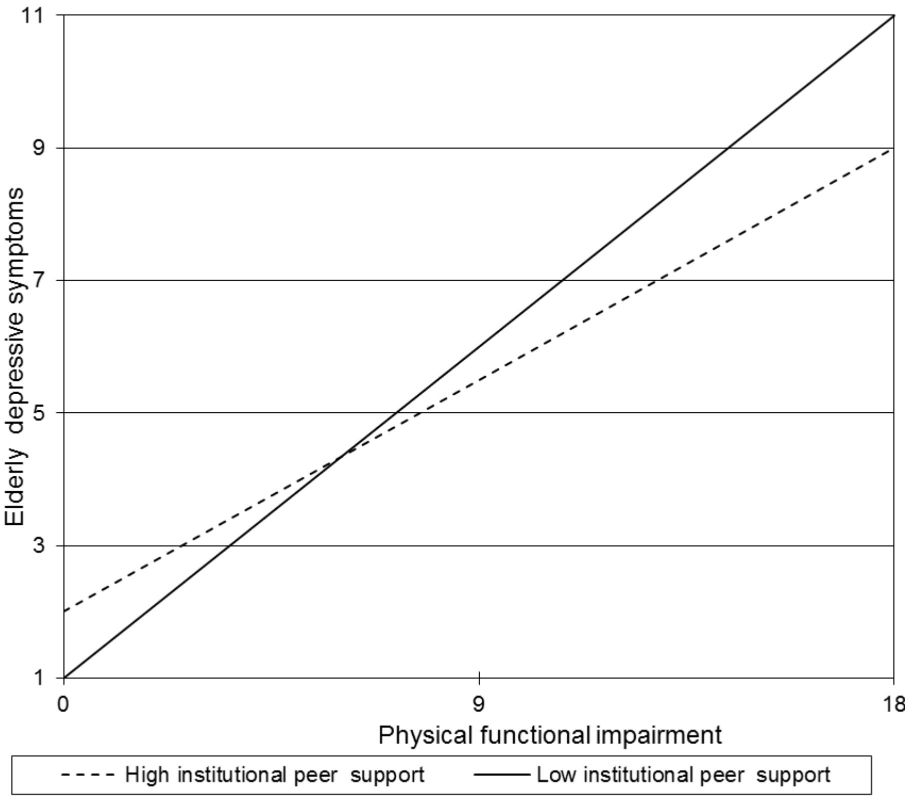

**FIGURE 1.** Effect of physical functional impairment on elderly depressive symptoms, moderated by perceived institutional peer support

**TABLE 2**
**Hierarchical Regression Analysis for the Prediction of Elderly Depression**

| Step | Predictor Variables | *B* | $R^2$ *Change* |
|---|---|---|---|
| 1 | Demographic characteristics | | |
| | Gender | -0.14* | 0.08*** |
| | Education | 0.23** | |
| 2 | Physical functional impairment | 0.11* | 0.39*** |
| | Perceived institutional peer support | -0.62*** | |
| | Perceived family support | -0.01 | |
| 3 | Interaction term | | |
| | Physical functional impairment × perceived institutional peer support | -0.16** | 0.03*** |
| | Physical functional impairment × perceived family support | 0.05 | |

*Note:* *B* = Standardized regression coefficient. **p* < 0.05; ***p* < 0.01; ****p* < 0.001.

### DISCUSSION

Results showed that the level of depressive symptoms of the elderly in the present study was relatively low, as compared to previous studies conducted in nursing homes in Hong Kong and the U.S. Such a discrepancy might be due to the fact that most of the participants in the present study had stayed in the nursing homes for a long period of time, i.e., more than 2 years. They were quite familiar with the living environment and might have regarded the nursing home as their second home. Therefore, this might lead to better psychological well-being and a lower level of depressive symptoms in the current sample. Findings indicated that female elders reported more depressive symptoms than male elders, supporting Hypothesis 1a. This coincides with both local and Western studies[30,18]. The biological, behavioral, and psychosocial responses to demands are different between men and women[31], which may explain the phenomenon of gender difference in depression. Although men and women experience the same stressors, women will have a higher sensitivity and are more responsive to stress, both biologically and psychologically[32], hence contributing to women's greater vulnerability to depression. Although it was hypothesized that elderly with higher education level would have a lower level of depressive symptoms, a contradictory result was found in the current study. Thus, Hypothesis 1b was not supported. It may be because the elderly with higher education levels have a higher expectation in later life; for example, be more contributive and live more independently. However, due to their health problems, they need to live in the nursing homes and receive intensive care. This unexpected institutionalization may affect their self-esteem and self-worth, thus contributing to more depressive symptoms.

Results showed that a higher level of functional impairment was related to a higher level of depressive symptoms. Hence, Hypothesis 2a was supported. In line with previous epidemiological and gerontological studies, functional impairment was related to an increase in depressive symptoms in old age[33,34]. Since most nursing home residents have a certain level of physical functional impairment, they need to receive different kinds of nursing care from the home staff. However, Chinese tradition emphasizes avoidance of getting help from others, except in very desperate situations. It is revealed in the Chinese saying, "It is better to help oneself rather than to request assistance from others." Therefore, being dependent on others would generate feelings of demoralization among the elderly, thus affecting their psychological well-being (e.g., sense of uselessness, hopelessness) and increasing their level of depressive symptoms.

Although Chinese elders emphasize the importance of emotional and instrumental support from family members, the findings indicated that there was no significant correlation between perceived family support and depressive symptoms. Thus, Hypothesis 2b was not supported. Most of the elders in nursing homes are moderately or severely deteriorated in self-care abilities, consequently requiring more intensive and professional care in daily living to supplement family support. Moreover, the physical distance from their family members and unavailability of immediate family assistance lessen the importance of family support. This explains why perceived family support is not a significant predictor of elderly depressive symptoms.

Results showed that a higher level of perceived institutional peer support was significantly correlated with a lower level of depressive symptoms, thus supporting Hypothesis 2c. This corresponds to previous studies indicating that the elderly who were embedded in active social networks tended to have a lower incidence of depression[35]. Institutional peer support gave the elderly a sense of cohesion and belongingness, leading to their psychological well-being and reducing their level of depression[36]. Since elders spend most of their time with their peers in the nursing home, they would have more opportunities for interaction and share similar circumstances and challenges. Hence, support from the peers provides a caring and supportive living environment, contributing to a decrease in the level of depressive symptoms. However, the result needs to be interpreted with caution because there are significant differences in perceived institutional peer support between the depressed and nondepressed samples. When elders are depressed, their perceptions of how much institutional peer support they receive may be negatively impacted by the depression. Hence, some participants' responses to the measures of perceived support may have reflected the impact of a depressed mood rather than the reality of support in their lives. Future studies with different methodologies, such as natural observation, and multiple informants may be needed for a more objective measurement of peer support.

With regard to the buffering effect, the findings indicated that perceived institutional peer support, but not perceived family support, moderated the effect of physical functional impairment on elderly depressive symptoms. Thus, Hypothesis 3 was partially supported, giving partial support to the stress-buffering model. The result is consistent with previous research indicating that satisfaction with social support buffered the adverse impact of disability on late-life depression[10]. As mentioned earlier, peers in the nursing homes can provide more immediate emotional and instrumental support to the elders with physical impairment, which may not be available from the family members due to the physical distance. They may also share their own experiences and act as models in combating functional impairment. When Chinese are in need of help, they usually find assistance from their neighbors more efficient than from the family members. This is reflected by a Chinese proverb, "Neighbors residing nearby are better than family members living far away from one another." The feeling of being supported may lessen the adverse impact of physical impairment, which in turn may lead to a lower level of depressive symptoms.

### IMPLICATIONS

Theoretically, findings of the current study partially support the stress-buffering model to include perceived institutional peer support as a coping resource that buffers the impact of physical stress on depressive symptoms. This study also has various practical implications. As perceived institutional peer support is a significant predictor and moderator of depressive symptoms, it may be helpful to organize support groups (e.g., mutual aid groups) and programs to promote mutual support among nursing home residents. Given that physical functional impairment is significantly related to depressive symptoms, it may also be helpful to maintain the physical functioning of the elderly by introducing training programs, such as rehabilitation exercise programs, to reduce their dependence and sense of worthlessness. In addition, counseling groups for unique target groups (e.g., females, elderly with higher education level) may be advantageous because homogeneous group members may be especially likely to have empathy for one another.

### LIMITATIONS

The present study has several limitations. First, findings cannot be generalized to all the elderly living in nursing homes due to convenience sampling. Second, more objective data can be collected from multiple informants who are familiar with the elderly. Third, the use of a cross-sectional design means that it is not possible to claim a causal relationship between the antecedents and elderly depression. Hence, a longitudinal research design can be used in future studies to investigate whether physical functional impairment or low level of perceived social support increases the severity of depressive symptoms.
